# Hybrid argon plasma coagulation (HybridAPC) versus sharp excision for the treatment of endometriosis: a prospective randomized clinical trial

**DOI:** 10.1007/s00404-022-06473-9

**Published:** 2022-03-13

**Authors:** Julia S. Keckstein, Simon Keckstein, Kristin Brunecker, Alexander Neugebauer, Daniela Nüssle, Sascha Hoffmann, Jürgen Andress, Felix Neis, Marcus Scharpf, Markus Enderle, Ralf Rothmund, Sara Y. Brucker, Martin Weiss Jun, Bernhard Kraemer

**Affiliations:** 1 Department of Women’s Health, Tuebingen University Hospital, Calwerstr. 7, 72076 Tübingen, Germany; 2 Department of Obstetrics and Gynecology, Klinikum Starnberg, Oßwaldstr. 1, 82319 Starnberg, Germany; 3 Department of Obstetrics and Gynecology, University Hospital, LMU Munich, Marchioninistr. 15, 81377 Munich, Germany; 4 Erbe Elektromedizin GmbH, Waldhoernlestr. 17, 72072 Tübingen, Germany; 5 Praxis Im Frauenzentrum Lindenhofspital, 3012 Bern, Switzerland

**Keywords:** Non-contact thermal ablation, Peritoneal endometriosis, Peritoneal adhesion formation, Waterjet

## Abstract

**Purpose:**

Endometriosis is a benign, but potentially serious gynaecological condition in terms of abdominal pain and impaired fertility. Laparoscopic excision techniques are considered the therapeutic standard. HybridAPC is presented as a novel technique for the non-contact thermal ablation of peritoneal endometriosis with simultaneous protection of the underlying thermosensitive structures by creating a needle-free elevated fluid cushion which enables a safer exposure and distance, as well as potentially improved peritoneal conditioning prior to APC.

**Methods:**

In this prospective randomized clinical trial, 39 patients with 132 superficial endometriotic lesions in total were treated with HybridAPC or sharp excision in an initial laparoscopic procedure according to randomization. In a second-look laparoscopy, adhesion formation was rated macroscopically. Histologic samples were taken from previously treated areas for evaluation of eradication rate.

**Results:**

The eradication rate was not significantly different between HybridAPC treatment and sharp excision (65 vs. 81%, *p* = .55). Adhesions formed in 5% of HybridAPC-treated lesions and in 10% after sharp excision (*p* = .49). HybridAPC treatment was significantly faster than sharp excision (69 vs. 106 s, *p* < .05). No intra- and postoperative complications were registered.

**Conclusion:**

This clinical trial demonstrates the feasibility of this novel surgical technique with a promising impact on adhesion prevention. Compared to sharp excision, HybridAPC is likely to be a safe, tissue-preserving, and fast method for the treatment of peritoneal endometriosis.

## Introduction

Endometriosis therapy focuses on pain relief, improvement of fertility or the prevention of organ dysfunction [1]. In addition to medical interventions, a laparoscopic approach is generally accepted as the mainstay to remove endometrial implants and restore anatomy [2, 3]. For peritoneal endometriosis, this is commonly achieved by different minimally invasive techniques such as sharp excision and subsequent haemostasis, ablation with monopolar or bipolar energy, CO_2_ laser vaporization, thermal destruction with helium plasma as well as ablation with plasma technology (e.g. PlasmaJet) [2, 4–6]. Possible frequent complications of direct or thermal peritoneal trauma are inflammatory reactions with subsequent adhesion formation of potentially excessive connective tissue bridges between abdominal or pelvic organs and the defect locations [7–9].

Two major aspects have an impact on surgical strategy and the choice of technology: peritoneal endometriosis can be located close to critical anatomical structures (ureter, bladder, bowel, nerves) that often lie directly under the layer of the endometriotic lesion. Secondly, the extent or depth of the lesion can grow deeper into the adjacent tissue as macroscopically assessed at first glance. Ideally, improved integrated technology would both eradicate peritoneal endometriosis with minimal mechanical or thermal trauma and preserve the underlying structures.

Non-contact *argon plasma coagulation* (APC) is a monopolar electrosurgical method where argon gas is partly ionized by a high voltage electrode, forming argon plasma between the active electrode and the tissue. The plasma thermally devitalizes the target tissue without direct contact, which possibly results in a reduced adhesion risk compared to contact coagulation methods such as monopolar coagulation, monopolar scissors, bipolar clamps and ultrasonic scalpel, strongly depending on the energy setting [10–12].

*HybridAPC* as a combination of needle-free waterjet injection and argon plasma is mainly used for treatment modalities in the GI tract [13–17]: a fine jet of water is first used to elevate the tissue (step 1) with the potential to protect the underlying structures from thermal energy of APC which can be applied afterwards through the same instrument (step 2).

The benefit of HybridAPC for peritoneal conditions with regard to the influence of electrosurgical trauma with minor adhesion formation due to positive effects of combined waterjet technology have been previously investigated by our group in an experimental animal model [18].

Consequently, the aim of this prospective randomized human study was to investigate the complete treatment of peritoneal endometriosis with HybridAPC in terms of eradication, application time, complications and adhesiogenesis in comparison to laparoscopic excision in a randomized prospective human study.

## Methods

This prospective, randomized, controlled, and single-blinded clinical study was conducted at the Department of Women's health at the University of Tuebingen, Germany, after approval by the Institutional Review Board (Ethics Committee of the Regional Board in Tuebingen, registration number 559/2015BO1) and registration in the German Registry of Clinical Trials (DRKS00011313). All included patients had given their informed consent in advance. The primary objective of the study was to compare the eradication rate of endometriosis after treatment with HybridAPC versus sharp excision in a laparoscopic setting. The rate of peritoneal adhesion formation and intervention time were evaluated as secondary objectives. To assess non-inferiority of HybridAPC towards sharp excision regarding the eradication rate, the number of cases was prospectively calculated and approved by the Department of Medical Biometry (University of Tuebingen, Germany). Randomization was done by assigning each pair of endometriotic lesions to one of the two possible treatment methods by a computer-generated randomization list.

**Fig. 1:**
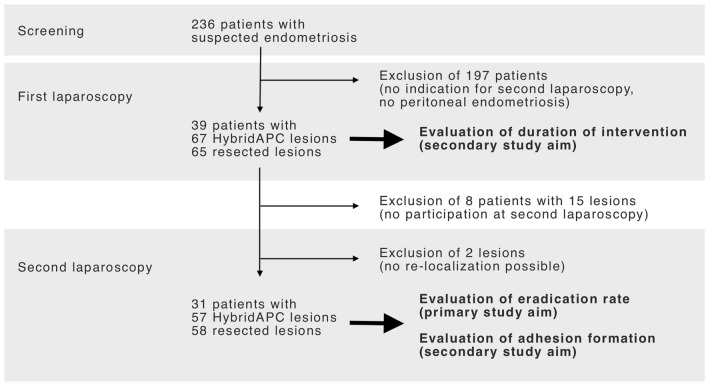
Flowchart of included and treated patients and lesions

The study consisted of two parts according to the study protocol (Fig. 1):

- Part 1: patients who presented with peritoneal endometriosis and an indication for a second laparoscopy, e.g. for further treatment of deep-infiltrating endometriosis (DIE) diagnosed during the first operation (according to the standard practice of our institution), were included. In part 1, peritoneal endometriosis was treated with HybridAPC (a) or excision (b) according to randomization of each single endometriotic lesion.
- Part 2: according to the protocol, a second-look laparoscopy was performed 4–12 weeks (1–3 cycle lengths) after the first procedure (part 1), and adhesion formation was macroscopically evaluated. The previously treated areas were excised for histopathological evaluation of residual endometriotic tissue, thus indicating complete or incomplete eradication.

### Part 1 (a) HybridAPC treatment of endometriotic lesions during first laparoscopy

**Fig. 2:**
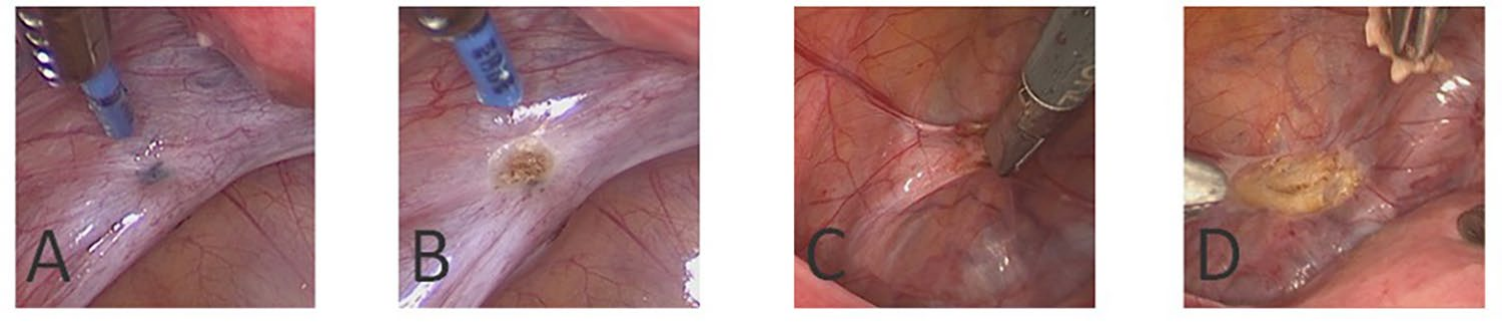
Peritoneal endometriosis before (**A**) and after (**B**) treatment with HybridAPC and before (**C**) and after (**D**) sharp excision

Generally, electrosurgery was deployed with a VIO300D generator (Erbe Elektromedizin GmbH, Germany). For the specific HybridAPC treatment of included lesions, an APC2 unit and a waterjet unit (ErbeJet2 waterjet surgery system, both Erbe Elektromedizin GmbH, Germany) were additionally used. The system is currently available with flexible probes only, requiring a standard tube as solid adapter through which the APC probe can be inserted into the trocar for laparoscopic use. For the peritoneal elevation of endometriotic lesions, a fluid saline cushion was generated by needleless waterjet injection (waterjet effect level 25), and the endometriotic tissue was ablated with non-contact argon plasma coagulation (PULSED APC E1, 25 W, argon flow 1 l/min) (Fig. 2).

### Part 1(b) sharp excision of endometriotic lesions during first laparoscopy

In the control group, endometriotic tissue was excised in a standard fashion using bipolar coagulation (BIPOLAR SOFT, effect 4, 60 W) for devitalization and scissors for tissue resection. All treated areas were photo documented for further assessment.

The excised lesions were embedded in paraffin and stained with haematoxylin and eosin. All specimens were investigated by a specialized pathologist (M.S.) to confirm endometriosis. In case of histomorphologic doubt, CD10 staining as immunohistochemical marker was used.

### Second-look laparoscopy

A second laparoscopy was performed ideally 4–12 weeks after the first intervention for the definite treatment of (deep-infiltrating) endometriosis. This two-stage intervention approach, which is part of our centre’s practice for possible DIE, was chosen for the study because it offers the possibility of a second look to both evaluate postsurgical adhesion formation and to control the success of the preceding endometriosis eradication according to the study protocol. The quantity and quality of peritoneal adhesions in the treated areas were analysed according to a previously published score [19]. Biopsies were taken from all treated areas and histologically investigated after blinding of the pathologist (M.S.) as mentioned above for remaining endometriosis.

### Statistics

The sample size was calculated based on the intended non-inferiority statement of HybridAPC towards sharp excision concerning the eradication rate of endometriosis. The following assumptions were made: eradication rate 90%, level of significance 5%, power 80%. The margin for a clinically non-relevant difference was set at *δ* = 15%. According to Bock [20], a sample size of *n* = 56 per group is required. Taking into account a drop out of 15% patients between first and second laparoscopy, 66 lesions per group and 132 lesions in total had to be included. As several lesions in one patient cannot be considered as independent from each other, models of logistic regression with random effects have been used.

For data analysis, the statistical software R was applied. Comparison between groups were performed by Fisher’s exact test, Barnard’s test, or Welch’s test. Non-inferiority of the HybridAPC was tested with a one-sided *Z* test; two-sided equivalence was tested with two one-sided tests (TOST).

## Results

A flowchart with the number of patients and endometriotic lesions is displayed in Fig. 1. Patients were 20–43 years old (median 32.8 years), body-mass-index average was 24.2 kg/m^2^, and 61% had an unfulfilled desire to conceive.

### First laparoscopy

At first laparoscopy, a total of 236 patients with suspected endometriosis were screened, from which 39 patients with both superficial and deep-infiltrating endometriosis could be included in the trial. 132 superficial endometriotic lesions (1 to 12 lesions per patient) were treated according to the study protocol with HybridAPC or sharp excision. Randomization of each lesion into one of the two treatment groups was conducted during surgery. 65 lesions were excised, and 67 lesions were treated with HybridAPC. Typical peritoneal endometriotic lesions before and after treatment are shown in Fig. 2. The first laparoscopies were performed by 11 laparoscopic surgeons, with the number of treated lesions per surgeon ranging from 2 to 52. No intra- and postoperative complications occurred in both interventional groups.

### Intervention time

**Fig. 3:**
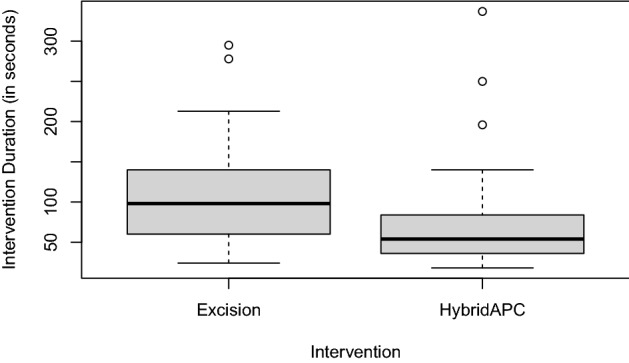
Intervention time in seconds for the procedures excision vs. HybridAPC

The mean intervention time with HybridAPC compared to excision during the first surgery was 69 vs. 106 s per lesion (*p* < 0.001) (Fig. 3).

### Second laparoscopy

The second laparoscopy was performed in 31 patients (79.5%) by eight surgeons. The median time between first and second laparoscopy was 8.4 weeks (5.2–38.7 weeks). 8 of 39 patients (15 lesions) dropped out after the first surgery as they refused further treatment. Two lesion sites had to be excluded from further analysis due to the impossibility of re-localization of the treated area during second laparoscopy.

### Histology-eradication rate

**Fig. 4:**
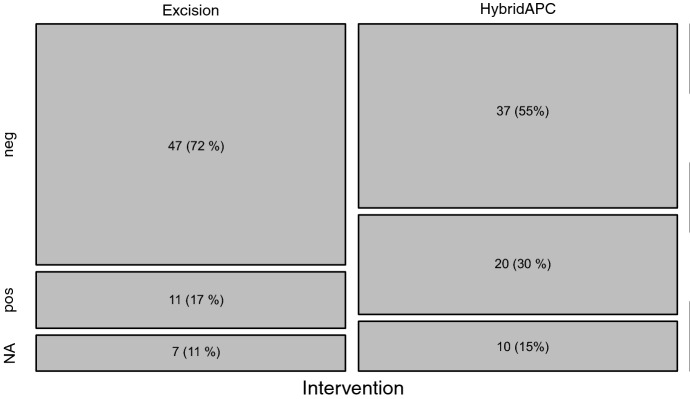
Eradication rate plotted against the type of intervention (absolute and relative numbers); *NA* not available value

The histological assessment of the resected tissue in the first operation revealed a rate of 15.4% (10/65 excised lesions) endometriosis-negative samples, which corresponds to a positive predictive value of 84.6%. After the second laparoscopy, 57 lesions in the HybridAPC group and 58 lesions in the excision group were eligible for histological assessment. The eradication rate (lesions without remaining endometriosis) was determined histologically and was found to be 64.9% (20/57 lesions) for HybridAPC and 81.0% (11/58 lesions) for excision (Fig. 4).

Using a TOST analysis, the difference between the two methods was − 0.161, 95%-CI (− 0.295, − 0.027), *p* = 0.55. HybridAPC is therefore not significantly non-inferior towards sharp excision. However, for a relevant inferiority of HybridAPC compared to sharp excision, using a two-sample binomial test, results were not significant, 95% CI (− 1, − 0.01), *p* = 0.082. To exclude an intra-individual dependence of the lesions, a random effects analysis was carried out which showed no dependence within a patient.

### Adhesions

Peritoneal adhesions were found in 9 out of 115 peritoneal lesions. The rate of adhesion formation was 5.3% (3/56 lesions) for HybridAPC and 10.5% (6/57 lesions) for sharp excision; 95% CI (0.414, 13.4419, *p* = 0.49. Table 1 summarizes the incidence of peritoneal adhesions.

**Table 1:** Absolute and relative incidence of adhesions

Intervention	Adhesions		
	No (%)	Yes (%)	NA
HybridAPC	53 (95)	3 (5)	11
Excision	51 (80)	6 (11)	8

*NA* not available value

## Discussion

Today, endometriosis is preferably treated laparoscopically. For the therapy of deep-infiltrating endometriosis, guidelines favour excision over ablative techniques [4]. However, regarding superficial peritoneal endometriosis, guidelines do not make a recommendation and studies that compare both methods (excision vs. ablation) are rare and remain inconclusive [21–23]. Suitable methods should combine effective treatment with low complication rates.

Argon plasma coagulation has been used in endometriosis therapy before, but the combination with injection capacity in one instrument is a novel technique, allowing a gentle and atraumatic separation of tissue planes [24]. Historically, a sharp injection cannula had to be used for this purpose, which poses the risk of organ injury. HybridAPC technology makes it possible to bluntly emit a saline jet, which perforates the peritoneum, building a fluid cushion underneath. The thermal penetration depth can be significantly reduced by the fluid cushion [11, 14, 18]. A desired thermal effect can hereby be concentrated on the surface, whilst the fluid cushion limits the risk of heat damage at depth. For application in the abdominal cavity where endometriotic lesions often are found close to delicate organs, such as the ureter, bladder or rectum, the fluid cushion could be a technique to avoid thermal damage to these sensitive structures.

As described above, there is still an ongoing debate about the optimal therapy for peritoneal endometriosis. In a previous study on rodent peritoneum, we demonstrated equality of APC and sharp excision regarding complete eradication [25]. This study seems to be one of the first in which the eradication rate of peritoneal endometriosis could be validated histologically by a second-look operation. In the present clinical trial, the eradication rate in the excision group was 81%, respectively, 65% in the HybridAPC group. If a relevant difference of *δ* = 0.15 is assumed, the statistical analysis suggests that the study objective of non-inferiority of HybridAPC to excision cannot be achieved significantly (*p* = 0.55). However, a relevant inferiority could not be demonstrated either (*p* = 0.082). Regarding eradication rate, this study did not yield statistical significance, indicating that HybridAPC is neither non-inferior nor significantly inferior towards sharp excision. Further studies with a larger study population are warranted to clarify this aspect conclusively.

**Fig. 5:**
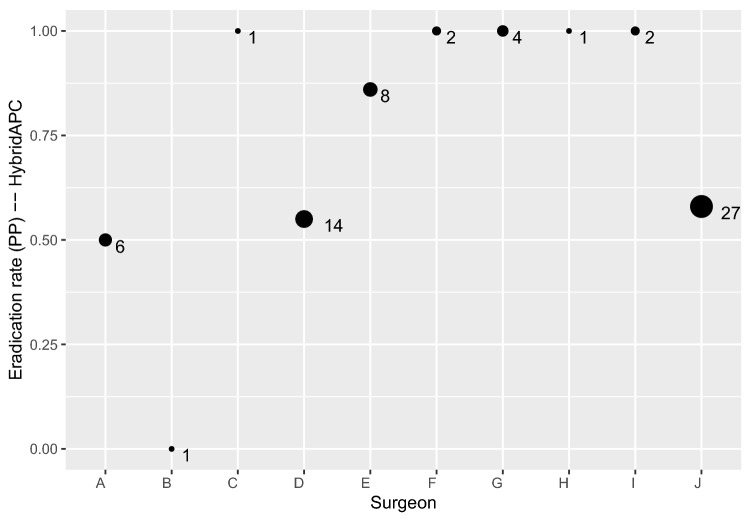
Eradication rates of HybridAPC per surgeon

**Fig. 6:**
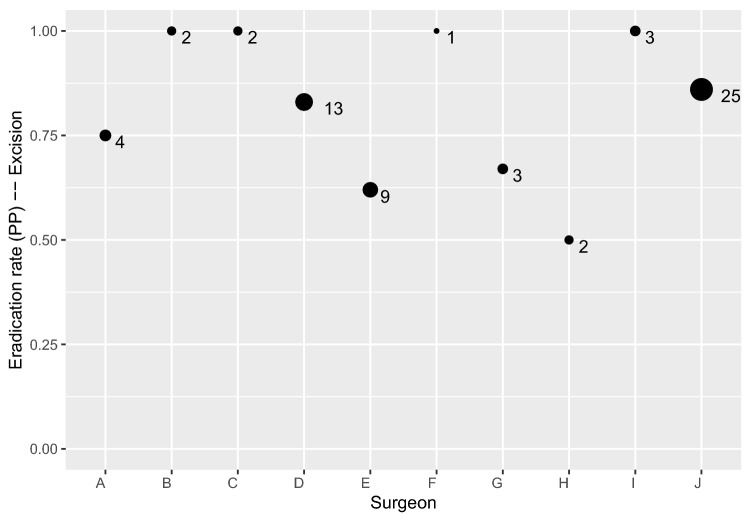
Eradication rates of excision per surgeon

Since the first laparoscopies were performed by a total of 11 surgeons, they inevitably went through different stages of a learning curve with HybridAPC application; therefore, the risk of undertreatment due to the surgeon’s individual risk assessment might play a role for the eradication rate in our study. However, the statistical analysis showed that the eradication rates of the individual surgeons were not significantly different, regardless of the number of treated lesions (Figs. 5 and 6). Because HybridAPC is most suitable for the treatment of superficial endometriosis, and these mild stages are not necessarily treated in specialized centres, this instrument might find its application area in an outpatient unit and not only in an endometriosis centre. Therefore, the fact that the surgeons in this study were not previously very frequent users of HybridAPC reduces the gap between study and real-world conditions. Yet, the eradication rates of sharp excision, being the standard method in our centre, and HybridAPC as a newly induced method, were comparable.

As adhesion formation can be a complication of endometriosis surgery, leading to subfertility, chronic pelvic pain and repeat laparoscopy [26, 27], the adhesiogenity of an operative technique is a relevant characteristic. In this present trial, HybridAPC led to fewer adhesions than excision of the endometriotic lesion, but the difference was not significant (5.3 vs. 10.5%). This complements the results from our study on a well-established rat peritoneum model: waterjet injection, to elevate a distinct area of peritoneum, does not tend to induce peritoneal adhesions [18]. Furthermore, argon plasma coagulation after waterjet elevation (HybridAPC) led to less adhesions than argon plasma coagulation (APC) alone, with only 2% versus 50% at the same energy intake in the before mentioned animal model [11, 18]. The reduction of adhesions might be affiliated to a moist environment which avoids desiccation of the peritoneum and offers a cooling effect by the fluid cushion [11].

With regard to intervention time, the use of HybridAPC is significantly faster than sharp excision. The mean difference between the two methods was 37 s (106 s for sharp excision versus HybridAPC 69 s). For sharp excision, instruments have to be changed for preparation, cutting and coagulation, often several times, which increases the risk of organ injuries as well as operating time [28]. With HybridAPC, all steps can be carried out with just one device, making instrument changes unnecessary. Operating time can be reduced, especially for disseminated or widespread superficial lesions, as the whole lesion area can be elevated with the waterjet, followed by devitalization of the endometriotic tissue with argon plasma. This aspect might be of particular interest in units with limited equipment or space for different generators.

The clinical outcome, such as improvement of life quality, pain relief and restoration of fertility, cannot be measured with this study design as both methods were applied in one patient. The effects of HybridAPC on symptomatology will need to be assessed in further studies.

## Conclusion

HybridAPC for gynaecological laparoscopy is a promising method to treat peritoneal endometriosis with the option of needle-free waterjet injection. Foremost it is a safe technique, which combines several advantages, such as easy and quick handling and a low adhesion rate.

## Data Availability

The dataset is available on Mendeley Data (https://data.mendeley.com/datasets/r7yrr6gkkc/1).
